# Supplementary material for: Hyperactivity of indirect pathway-projecting spiny projection neurons promotes compulsive behavior
Source: Nat Commun. 2024 May 24;15:4434. doi: 10.1038/s41467-024-48331-z (PMC11126597; doi:10.1038/s41467-024-48331-z)
Supplement: Supplementary file 1 — Supplementary Information [file 41467_2024_48331_MOESM1_ESM.pdf]

Hyperactivity of indirect pathway-projecting spiny projection neurons promotes compulsive behavior

Sean C Piantadosi*[1,2,3], Elizabeth E Manning*[2,4], Brittany L Chamberlain*[1,2], James Hyde[2,5], Zoe LaPalombara[1,2], Nicholas M Bannon[2], Jamie L Pierson[2], Vijay MK Namboodiri[6], Susanne E Ahmari[1,2#]

Supplementary Information

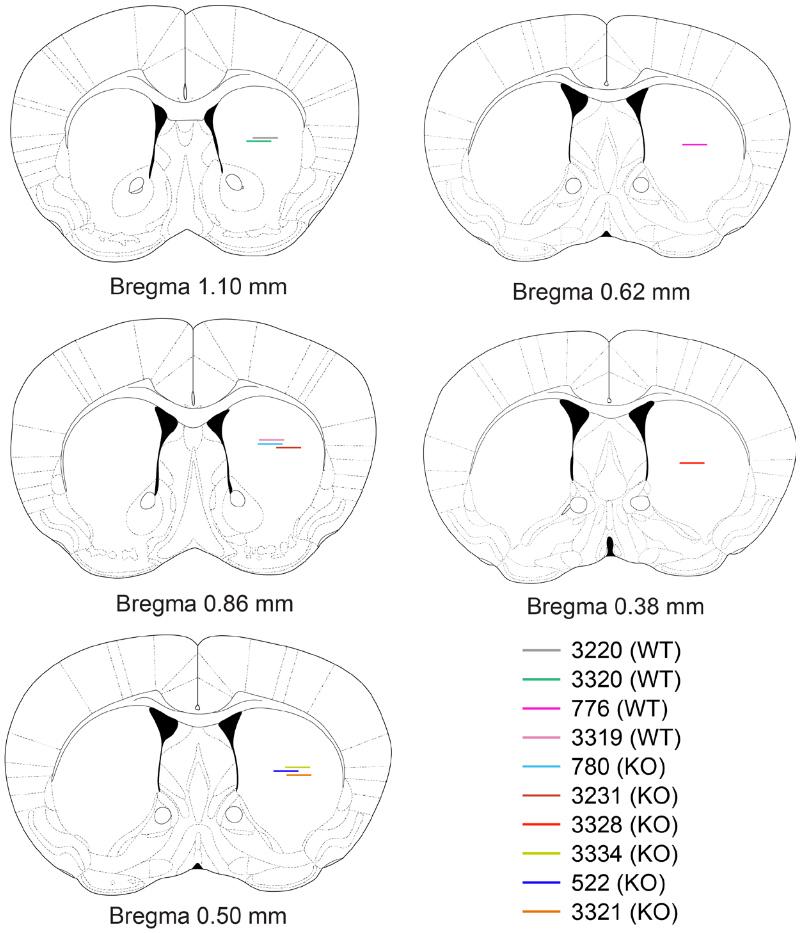

Supplementary Figure 1

Supplementary Figure 1. Extended data from CS calcium imaging. Lens placements in CS for subset of mice included in **Figure 1**. Individual horizontal lines indicate bottom of GRIN lens for a single mouse. Brain atlas overlay used with permission of Elsevier Science and Technology Journals from Paxinos and Franklin's the Mouse Brain in Stereotaxic Coordinates, Franklin Keith B.J., Paxinos, George, volume 5, copyright year 2019; permission conveyed through Copyright Clearance Center, Inc.

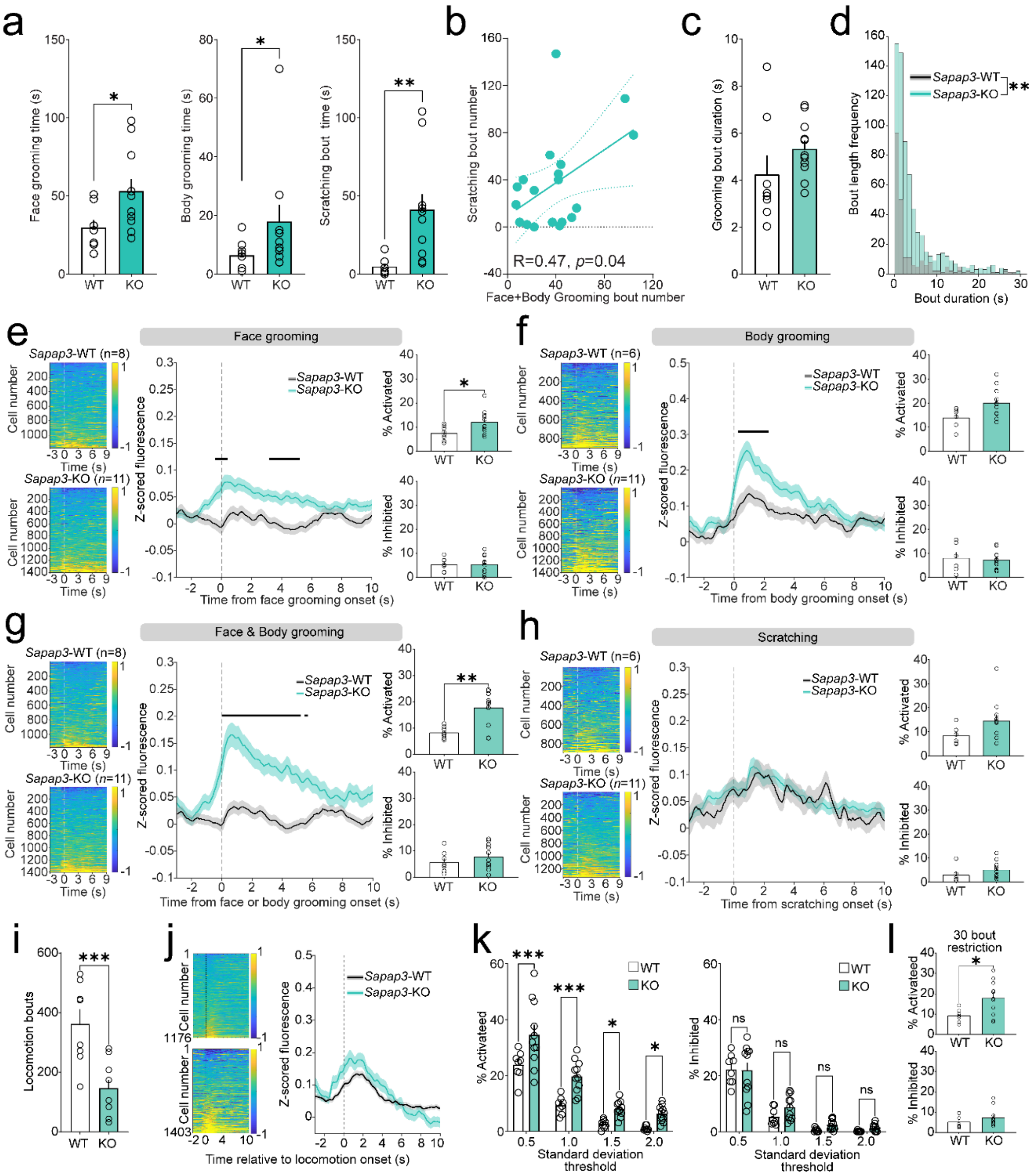

Supplementary Figure 2

Supplementary Figure 2. Extended data from CS calcium imaging. (**a**) Number of grooming bouts by subtype. *Sapap3*-KOs engage in a greater number of face grooming (two-tailed Mann Whitney Test, *U*=17, *p*=0.024), body grooming (*U*=15.5, *p*=0.02), and hind leg scratching (*U*=4.5, *p*=0.0004) bouts than -WTs. (**b**) A significant positive correlation was observed between the sum of face and body grooming bouts and the number of hind leg scratching bouts in WT and KO mice (*R*=0.47, *p*=0.04). (**c**) No difference in grooming bout duration between *Sapap3*-KOs and -WTs [*t*(18)=1.37, *p*=0.19]. (**d**) Histogram of grooming bout length frequency in *Sapap3*-KOs (teal) and -WTs (black) (Kolmogorov-Smirnov Test *D*=0.23, *p*<0.001). (**e,** *Left*) Heatmaps aligned to face-grooming bout onset in WTs (top) and KOs (bottom). (*Center*) Average Z-scored fluorescence aligned to face-grooming bout onset in WT (black) and KO (teal) mice. Black bar indicates significant time samples (*p*≤0.00038). (*Right*) Percentage of neurons activated [*t*(17)=2.4, *p*=0.03] and inhibited (*p*=0.90) during face-

grooming in WTs and KOs. (**f,** *Left*) Heatmaps aligned to body-grooming bout onset in WTs (top) and KOs (bottom). (*Center*) Average Z-scored fluorescence aligned to body-grooming bout onset in WT (black) and KO (teal) mice. Black bar indicates significant time samples ($p \leq 0.00038$). (*Right*) Percentage of neurons activated (two-tailed Mann-Whitney Test; U=14, p=0.06) and inhibited (p=0.89) during body grooming in WTs and KOs. (**g,** *Left*) Heatmaps aligned to the start of face- or body-grooming bouts in WTs (top) and KOs (bottom). (*Center*) Average Z-scored fluorescence aligned to body-grooming bout onset in WT (black) and KO (teal) mice. Black bar indicates significant time samples (two-tailed unpaired t-test, Bonferonni correction, $p \leq 0.00038$). (*Right*) Percentage of neurons activated [$t(17)$=3,76, p=0.001] and inhibited (p=0.33) during face and body grooming in WTs and KOs. (**h,** *Left*) Heatmaps aligned to the start of bouts of hind leg scratching in WTs (top) and KOs (bottom). (*Center*) Average Z-scored fluorescence aligned to hind-leg-scratching-bout onset in WT (black) and KO (teal) mice (right, $p \geq 0.00038$). (*Right*) Percentage of neurons activated (two-tailed Mann-Whitney Test; U=16, p=0.09) and inhibited (Mann-Whitney Test; U=16, p=0.09) during hind leg scratching in WTs and KOs. (**i**) *Sapap3*-KOs engage in fewer bouts of locomotion relative to WT littermates [$t(17)$=4.21, p=0.0006]. (**j**) Locomotion-start aligned calcium fluorescence averaged across trials from CS SPNs in WT (top) and KO (bottom) mice. Locomotion-start aligned calcium activity is not significantly different in WT and KO mice (right, $p \geq 0.00038$). (**k**; *Left*) Proportion of activated neurons classified at multiple standard deviation thresholds (δ=0.5, 1.0, 1.5, 2.0). Two-way ANOVA: significant main effect of genotype [$F(1,17)$=15.3, p=0.0011], significant main effect of threshold [$F(3,51)$=152.1, $p<0.0001$], with no significant interaction between genotype and threshold [$F(3,51)$=2.5, p=0.07], indicating that the proportion of activated SPNs was elevated in KOs regardless of classification threshold. To confirm this, we conducted non-parametric two-tailed Mann-Whitney Tests between genotype for each threshold, finding significantly increased activated SPN proportion at 2.0 SD (U=2, p=0.001), 1.5 SD (U=4, p=0.003), 1.0 SD (U=3, p=0.002), and 0.5 SD (U=16, p=0.02). (**k**; *Right*) Proportion of inhibited neurons classified at multiple standard deviation thresholds (δ=0.5, 1.0, 1.5, 2.0). Two-way ANOVA: no significant main effect of genotype [$F(1,17)$=1.15, p=0.29], significant main effect of threshold [$F(3,51)$=147.0, $p<0.0001$], and no significant interaction between genotype and threshold [$F(3,51)$=0.91, p=0.44], indicating that there was no significant difference between WTs and KOs in the proportion of inhibited neurons at any threshold. (**l**) Proportion of grooming-selective neurons identified in WTs and KOs when grooming bouts were capped to mean number of WT grooming bouts (30 bouts). Compared to WTs, KOs still had significantly greater proportions of grooming-onset activated neurons ($t(17)$=2.84, p=0.01). No differences in grooming-onset inhibited neurons were identified (p=0.24). Data are presented as mean values +/- SEM. Source data are provided as a Source Data file.

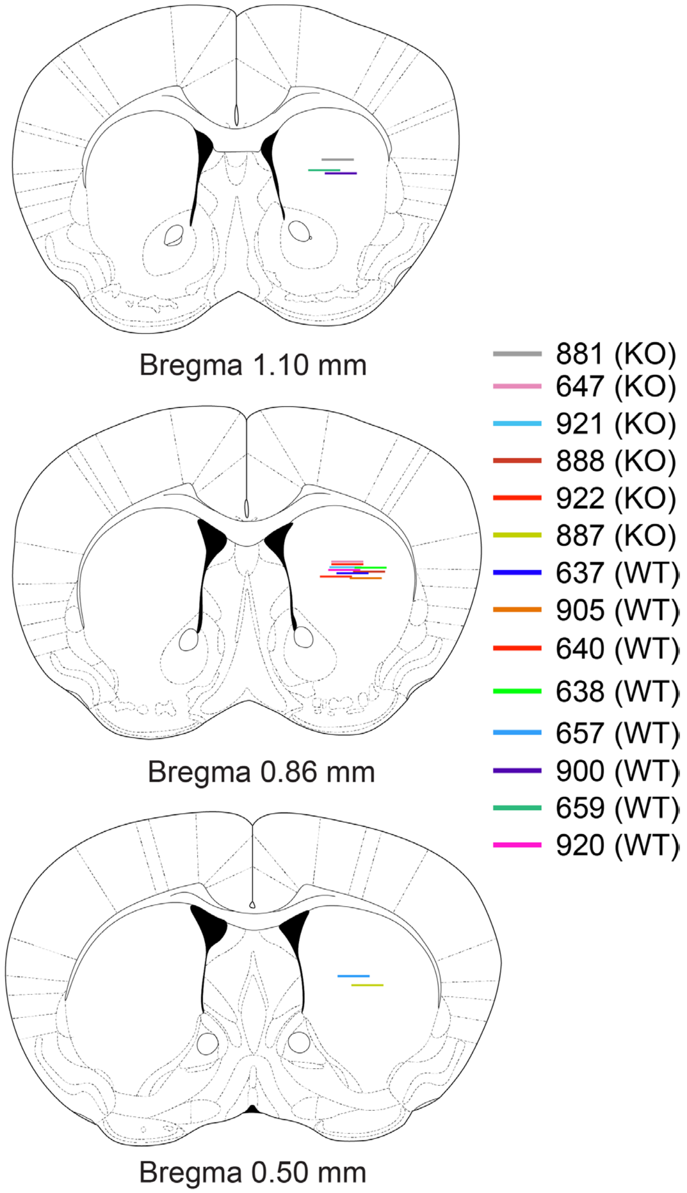

Supplementary Figure 3

Supplementary Figure 3. Lens placement for D1-Cre / *Sapap3*-KO and -WT imaging. Histological verification of GRIN lens placements in CS. Individual horizontal lines indicate bottom of GRIN lens for a single mouse. Brain atlas overlay used with permission of Elsevier Science and Technology Journals from Paxinos and Franklin's the Mouse Brain in Stereotaxic Coordinates, Franklin Keith B.J., Paxinos, George, volume 5, copyright year 2019; permission conveyed through Copyright Clearance Center, Inc.

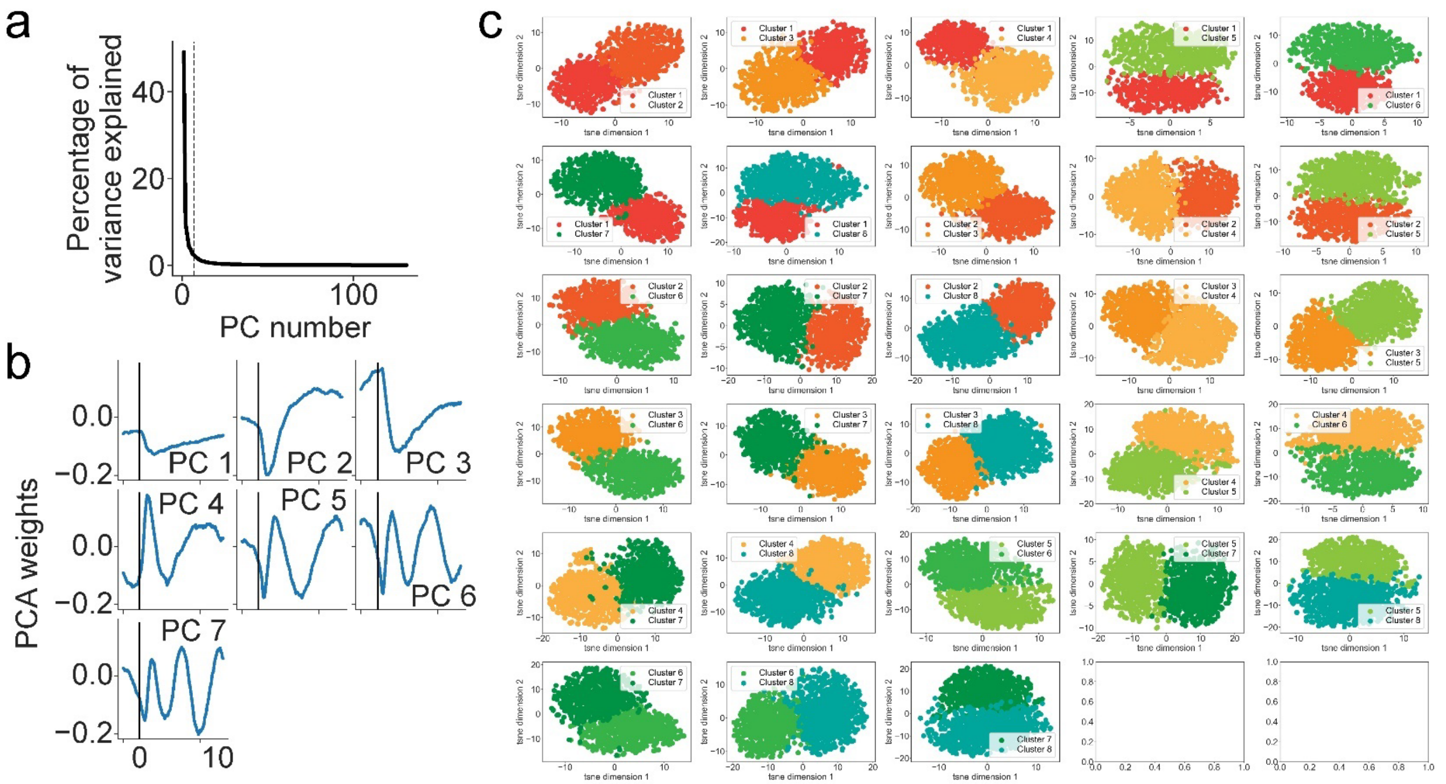

Supplementary Figure 4

Supplementary Figure 4. Spectral clustering analysis of central striatal SPNs. (**a**) Scree plot of percentage of variance explained per principal component; dotted line indicates number of principal components retained (7). (**b**) Plots of individual retained components. (**c**) Cluster separation in activity space. Neurons belonging to each pair of clusters are shown in a 2D t-distributed Stochastic Neighbor Embedding (t-SNE) plot to show that each cluster is separable from other clusters. Perplexity was set to 100 for this visualization.

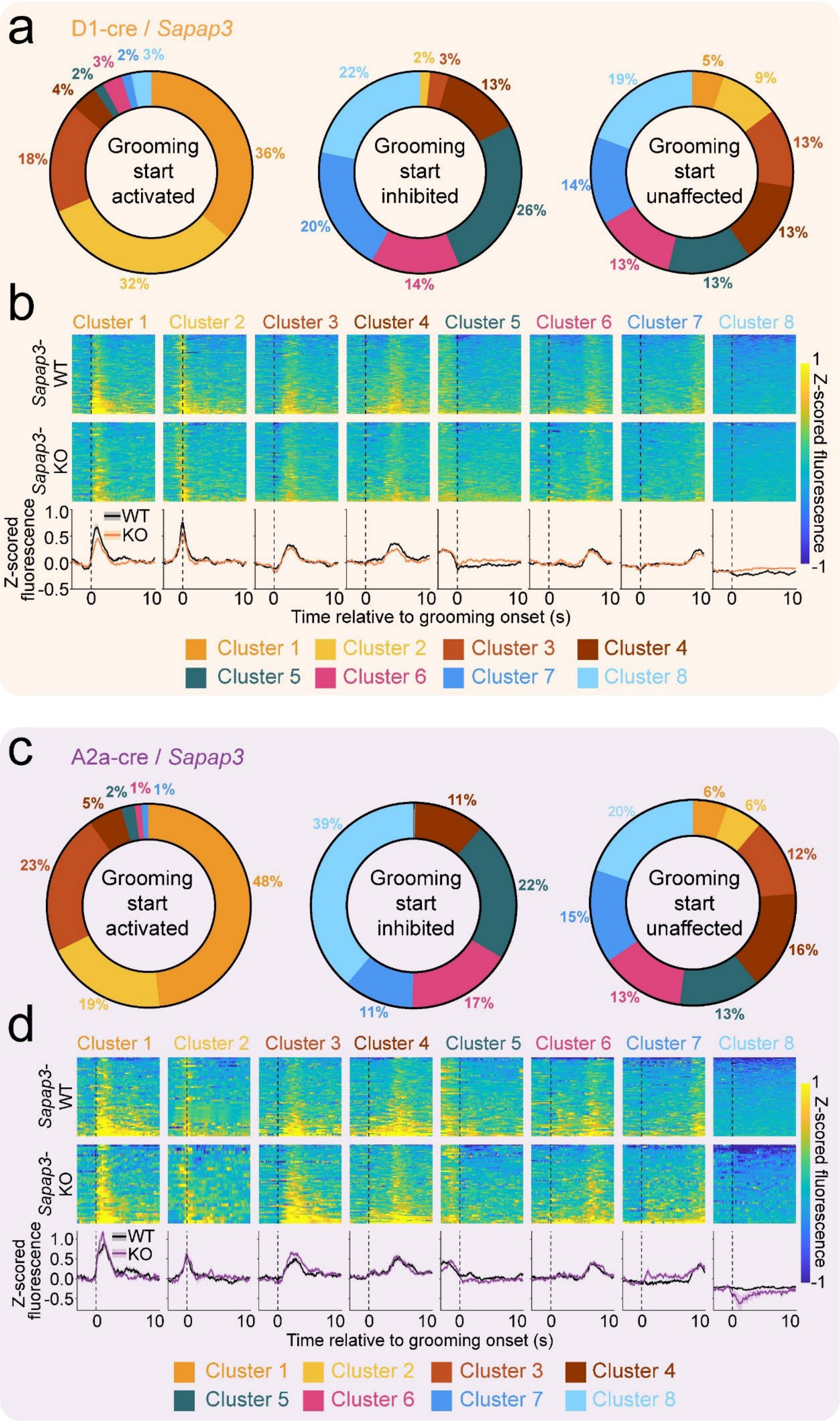

Supplementary Figure 5

Supplementary Figure 5. Overlap between spectral clustering and classifier selectivity in D1-Cre and A2a-Cre WTs and KOs. (**a**) Percentage overlap between (1) neurons identified as grooming start-activated (left), grooming start-inhibited (center), and grooming start-unaffected (right) (**Fig.2f**) by our statistical classification, and (2) cluster identity of these neurons as determined by spectral clustering for WT and KO D1-SPNs. Color indicates cluster identity, and the percentage indicates overlap with grooming-start-modulated neurons. (**b**) Heatmaps and mean traces replotted from **Fig.2i** for each functional cluster for reference. (**c**) Percentage overlap between (1) neurons identified as grooming start-activated (left), grooming start-inhibited (center), and grooming start unaffected (right) (**Fig.3f**) by our statistical classification, and (2) cluster identity of these neurons as determined by spectral clustering for WT and KO D2-SPNs (A2a-Cre). Color indicates cluster identity, and the percentage indicates overlap with grooming-start-modulated neurons. (**d**) Heatmaps and mean traces replotted from **Fig.3i** for each functional cluster for reference. Data are presented as mean values +/- SEM. Source data are provided as a Source Data file.

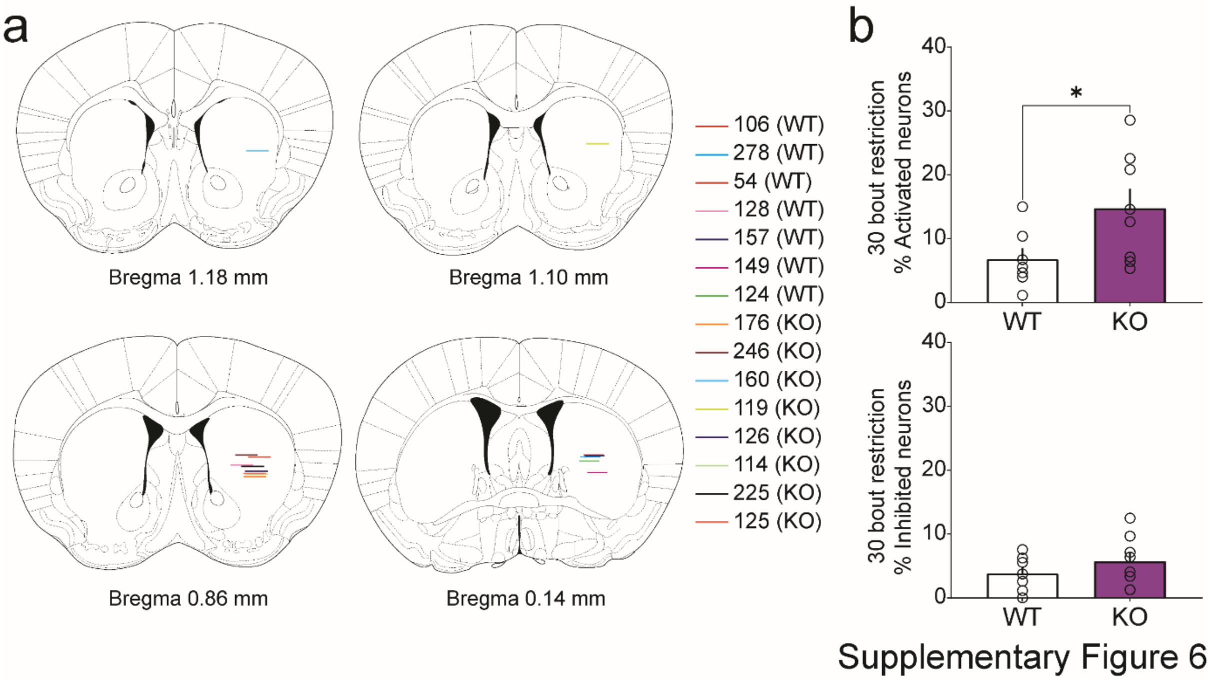

Supplementary Figure 6. Additional data for A2a-Cre$^{+/-}$ / *Sapap3*-KO and -WT imaging. (**a**) Histological verification of GRIN lens placements in CS. Individual horizontal lines indicate bottom of GRIN lens for a single mouse. Brain atlas overlay used with permission of Elsevier Science and Technology Journals from Paxinos and Franklin's the Mouse Brain in Stereotaxic Coordinates, Franklin Keith B.J., Paxinos, George, volume 5, copyright year 2019; permission conveyed through Copyright Clearance Center, Inc. (**b**) Percentage of grooming-onset activated and grooming-onset inhibited neurons identified when the number of grooming bouts for analysis was restricted to the mean number of grooming bouts engaged in by WT mice (30 bouts). *Sapap3*-KO mice have a greater proportion of D2-SPNs activated at the onset of grooming than -WTs (two-tailed Mann-Whitney test, U=11, p=0.03). No difference in the percentage of grooming-onset inhibited D2-SPNs was identified (p=0.17). Data are presented as mean values +/- SEM**.** Source data are provided as a Source Data file.

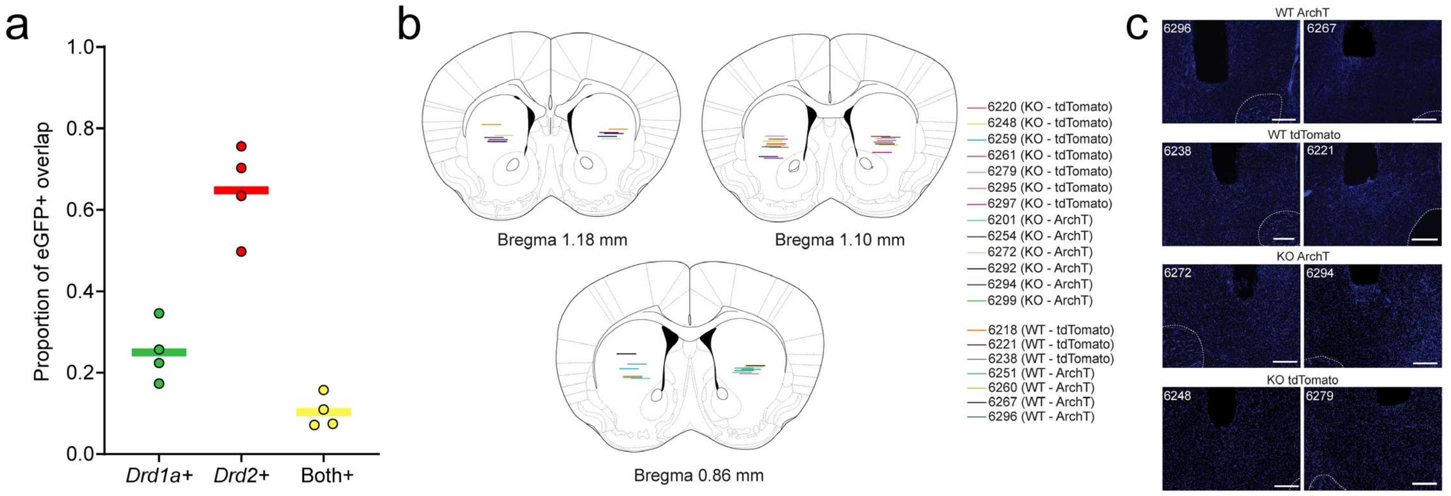

Supplementary Figure 7

Supplementary Figure 7. Retro-GPe *in situ* validation and fiberoptic implant histology. (**a**) Proportion of eGFP+ SPNs in the central striatum expressing *Drd1a* (green), *Drd2* (red), or both *Drd1a* and *Drd2* (yellow). (**b**) Histological verification of bilateral optic fiber placements in CS of KO (*n*=13) and WT (*n*=7) mice. 5 KOs and 1 WT were excluded due to fiber localization outside of CS. Individual horizontal lines indicate bottom of optic fiber for a single mouse. Brain atlas overlay used with permission of Elsevier Science and Technology Journals from Paxinos and Franklin's the Mouse Brain in Stereotaxic Coordinates, Franklin Keith B.J., Paxinos, George, volume 5, copyright year 2019; permission conveyed through Copyright Clearance Center, Inc. (**c**) Representative histological images and lens tracks from 2 mice for each genotype and treatment (2 WT ArchT, 2 WT tdTomato, 2 KO ArchT, 2 KO tdTomato). Source data are provided as a Source Data file.

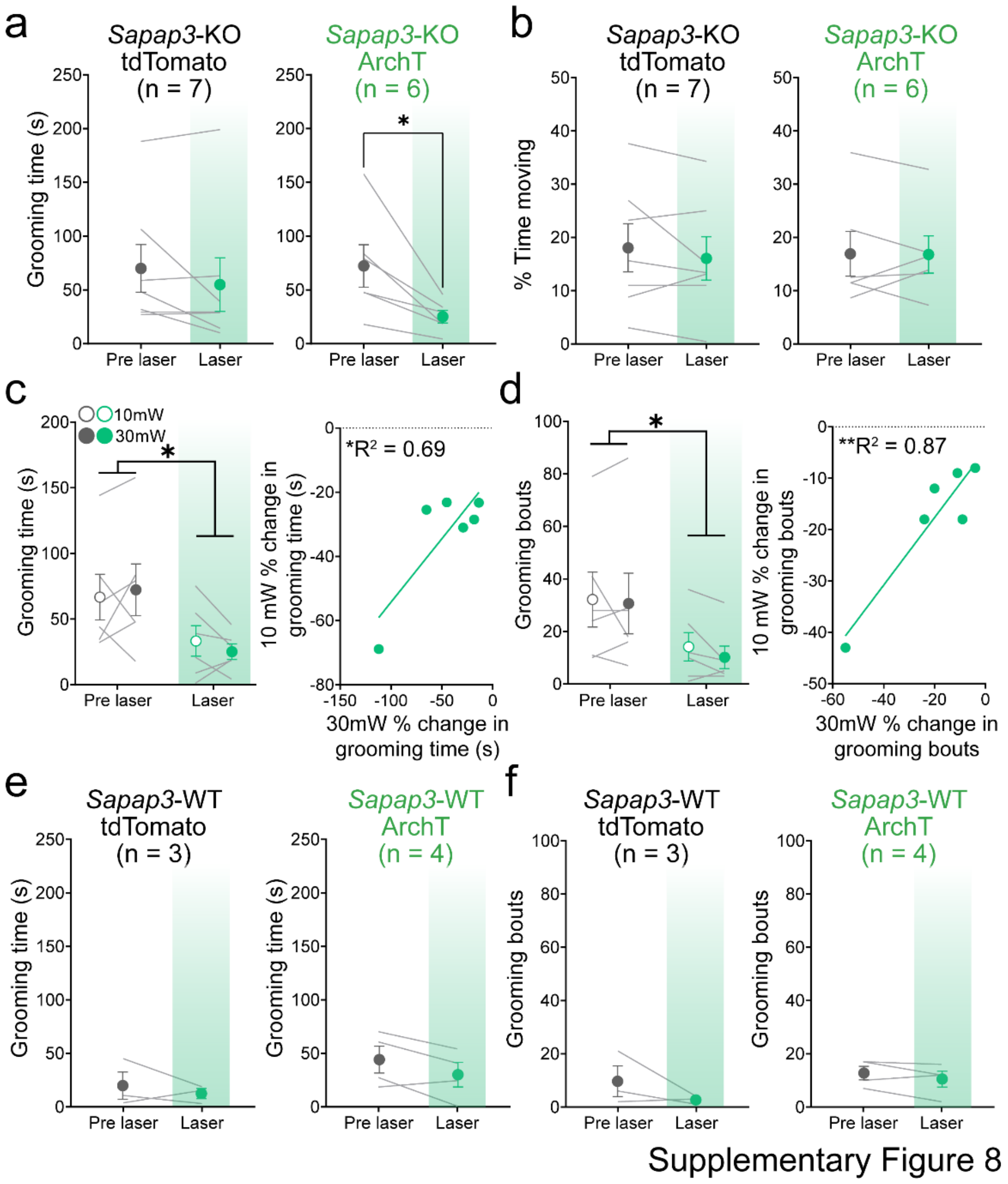

Supplementary Figure 8. Inhibition of striatopallidal SPNs. (**a**) No effect of laser illumination on time spent grooming in tdTomato-expressing KO mice (left; Two-sided Wilcoxon matched-pairs signed rank test, *p*=0.58). Significant reduction in time spent grooming during laser illumination in ArchT-expressing KO mice (Two-sided Wilcoxon matched-pairs signed rank test *p*=0.03). (**b**) No effect of laser illumination on percentage of total time spent moving compared to pre-laser time in either tdTomato- (left) or ArchT-expressing (right) KO mice (Two-sided Wilcoxon matched-pairs signed rank test, all *p*=0.47 ArchT and *p*=0.84 tdTomato). (**c**; *left*) Grooming time in *Sapap3*-KO mice expressing ArchT is reduced during laser illumination periods from sessions with 10mW laser power (open circles) and 30mW laser power (closed circles). Two-way repeated measures ANOVA, main effect of laser on/off [$F(1,5)=14.12$, $p=0.013$], no main effect of laser power [$F(1,5)=0.02$, $p=0.89$], no interaction between laser on/off and laser power [$F(1,5)=1.9$, $p=0.23$]. (**c**; *right*) Correlation between the laser-induced change in grooming time for 10mW and 30mW of laser power in *Sapap3*-KO mice expressing ArchT (Pearson correlation coefficient $R^2=0.69$, $p=0.04$). (**d**; *left*) Grooming bout number in *Sapap3*-KO mice expressing ArchT is reduced during laser

illumination periods from sessions with 10mW laser power (open circles) and 30mW laser power (closed circles). Two-way repeated measures ANOVA, main effect of laser on/off [$F(1,5)=9.3$, $p=0.03$], no main effect of laser power [$F(1,5)=0.67$, $p=0.45$], no interaction between laser on/off and laser power [$F(1,5)=61$, $p=0.47$]. (**d**; *right*) Correlation between the laser-induced change in grooming bout number for 10mW and 30mW of laser power in *Sapap3*-KO mice expressing ArchT (Pearson correlation coefficient $R^2=0.87$, $p=0.007$). (**e**) No effect of laser illumination on time spent grooming in tdTomato- (left) or ArchT-expressing (right) WT littermates (Two-sided Wilcoxon matched-pairs signed rank test, all $p=0.75$ tdTomato, $p=0.25$ ArchT). (**f**) No effect of laser illumination on the number of grooming bouts in tdTomato- (left) or ArchT-expressing (right) WT littermates (Two-sided Wilcoxon matched-pairs signed rank test, all $p=0.50$ tdTomato, $p=0.38$ ArchT). Green shading indicates laser illumination. Data are presented as mean values +/- SEM**.** Source data are provided as a Source Data file.

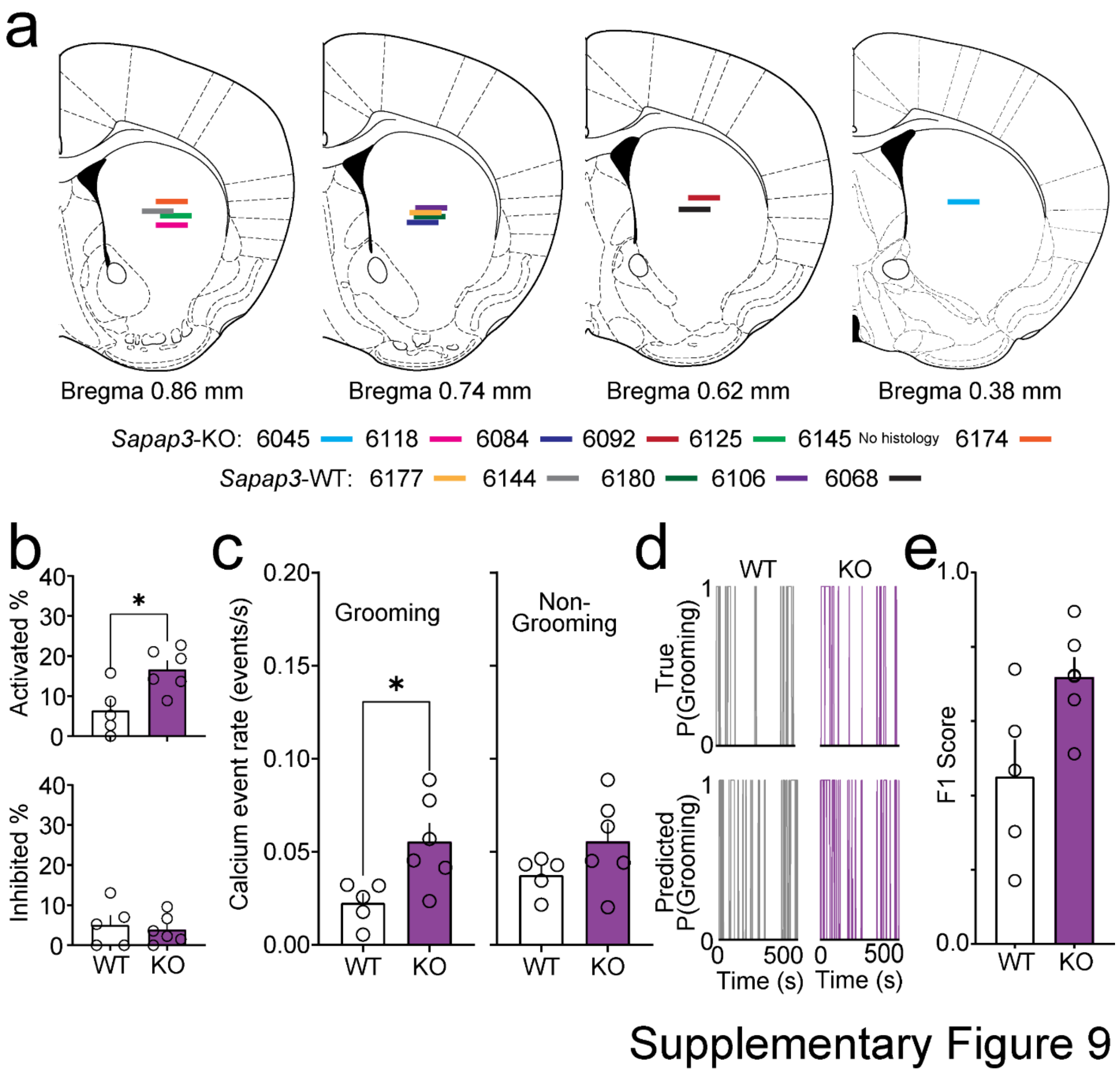

Supplementary Figure 9. Imaging striatopallidal SPNs in WT and KO mice. (**a**) Histological verification of GRIN lens placements in CS of WT (*n*=5: 5 female) and KO (*n*=6: 6 female) mice. Brain atlas overlay used with permission of Elsevier Science and Technology Journals from Paxinos and Franklin's the Mouse Brain in Stereotaxic Coordinates, Franklin Keith B.J., Paxinos, George, volume 5, copyright year 2019; permission conveyed through Copyright Clearance Center, Inc. (**b**) *Sapap3*-KOs have a greater proportion of groom-onset activated striatopallidal SPNs than WTs (two-tailed Mann-Whitney test, U=3, p=0.03), with no changes in the proportion of inhibited neurons (p=0.93). (**c**) Elevated calcium event rates during grooming (left) but not during non-grooming periods (right) in KO mice relative to WTs. (**d**) Example of RUSBoost classification of grooming probability in WT (grey, left) and KO (purple, right) based on striatopallidal population activity. (**e**) No significant difference in F1 score was identified between WTs and KOs (two-tailed Mann-Whitney test, U=5, p=0.08). $^{*}p<0.05$, $^{\#}p<0.1$. Data are presented as mean values +/- SEM. Source data are provided as a Source Data file.

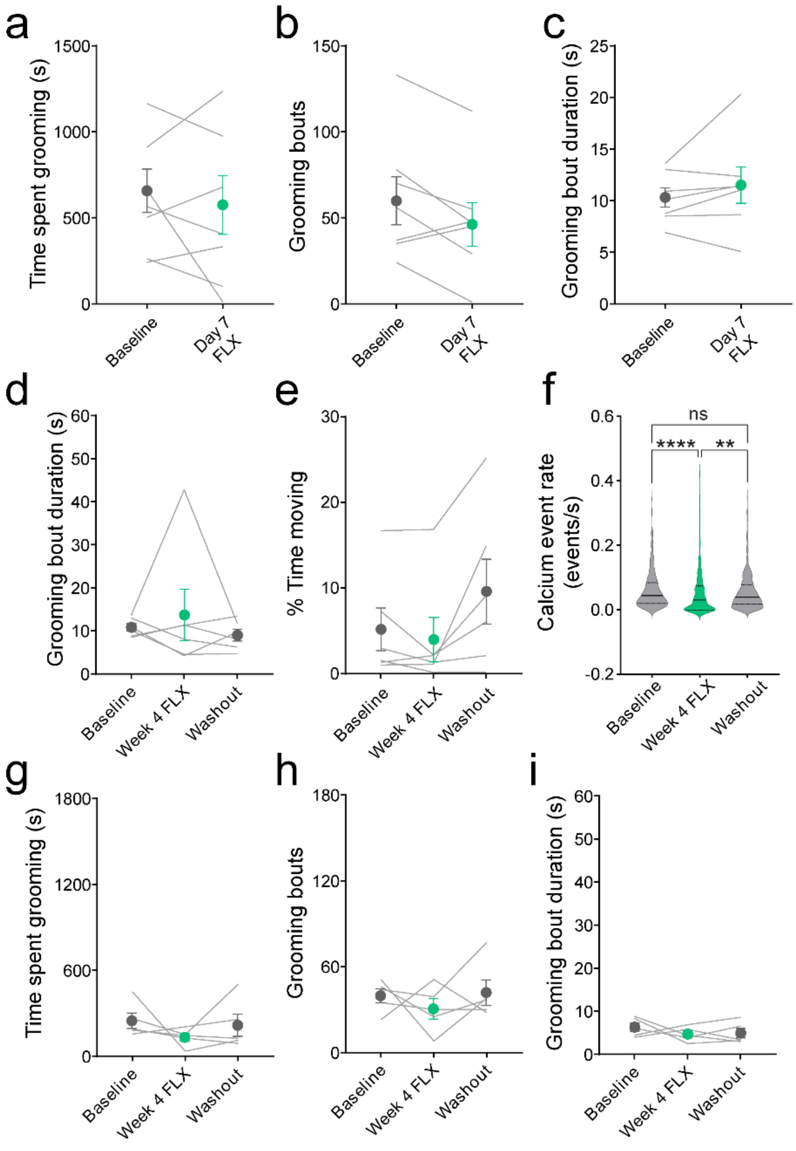

Supplementary Figure 10

Supplementary Figure 10. Fluoxetine treatment in striatopallidal imaging WT and KO mice. (**a**) 7 days of intraperitoneal fluoxetine (FLX; 5 mg/kg) treatment did not affect time spent grooming (two-sided Wilcoxon matched-pairs signed rank test, p=0.69), (**b**) number of grooming bouts (two-sided Wilcoxon matched-pairs signed rank test, p=0.08) or (**c**) grooming bout duration (two-sided Wilcoxon matched-pairs signed rank test p=0.38) in *Sapap3*-KOs. (**d**) Chronic fluoxetine treatment in drinking water (18mg/kg) did not affect grooming bout duration [(Friedman test ($Fr(3,6)$=1.3,p=0.57)] or (**e**) percentage of time spent moving during a 40 minute session [(Friedman test ($Fr(3,6)$=3,p=0.25)]. (**f**) Effect of chronic fluoxetine on calcium event rate of individual striatopallidal SPNs (Kruskal-Wallis ANOVA, H=23.29, p<0.0001). 18 mg/kg chronic fluoxetine in the drinking water significantly reduced striatopallidal calcium event rate compared to baseline (Dunn's multiple comparison test, Z=4.77, p<0.0001) and washout periods (Dunn's multiple comparison test, Z=3.12, p=0.01). No effect of chronic fluoxetine on (**g**) time spent grooming [(Friedman test ($Fr(3,5)$=1.6,p=0.52)], (**h**) number of grooming bouts

[(Friedman test ($Fr(3,5)=0.4, p=0.95$)], or (**i**) grooming bout duration in WT littermates [(Friedman test ($Fr(3,6)=1.3, p=0.57$)]

. Data are presented as mean values +/- SEM. Source data are provided as a Source Data file.
