# Supplementary material for: Hyperactivity of indirect pathway-projecting spiny projection neurons promotes compulsive behavior
Source: Nat Commun. 2024 May 24;15:4434. doi: 10.1038/s41467-024-48331-z (PMC11126597; doi:10.1038/s41467-024-48331-z)
Supplement: Supplementary file 3 — Description of Additional Supplementary Files [file 41467_2024_48331_MOESM3_ESM.pdf]

Description of Additional Supplementary Files

File name: Supplementary Data 1
Description: Table containing all RUSBoost classifier metrics for all datasets.

File name: Supplementary Movie 1
Description: Raw fluorescence recording (left) and CNMFe extracted signal (right) of central striatal neurons from a representative mouse recorded with hsyn-GCaMP6m during a baseline grooming session. Video is sped up 8x (total recording time = 40 minutes).

File name: Supplementary Movie 2
Description: Raw fluorescence recording (left) and CNMFe extracted signal (right) of central striatal neurons from a representative mouse recorded with DIO-GCaMP6m in a D1-Cre mouse during a baseline grooming session. Video is sped up 8x (total recording time = 40 minutes).

File name: Supplementary Movie 3
Description: Raw fluorescence recording (left) and CNMFe extracted signal (right) of central striatal neurons from a representative mouse recorded with DIO-GCaMP6m in a A2a-Cre mouse during a baseline grooming session. Video is sped up 8x (total recording time = 40 minutes).

File name: Supplementary Movie 4
Description: Raw fluorescence recording (left) and CNMFe extracted signal (right) of central striatal neurons from a representative mouse recorded with DIO-GCaMP6m in a WT mouse injected with a DIO-Cre virus into the GPe during a baseline grooming session. Video is sped up 8x (total recording time = 40 minutes).
