# Supplementary material for: Hyperactivity of indirect pathway-projecting spiny projection neurons promotes compulsive behavior
Source: Nat Commun. 2024 May 24;15:4434. doi: 10.1038/s41467-024-48331-z (PMC11126597; doi:10.1038/s41467-024-48331-z)
Supplement: Supplementary file 9 — Reporting Summary [file 41467_2024_48331_MOESM9_ESM.pdf]

nature portfolio

Corresponding author(s): Susanne E Ahmari

Last updated by author(s): Mar 8, 2024

Reporting Summary

Nature Portfolio wishes to improve the reproducibility of the work that we publish. This form provides structure for consistency and transparency in reporting. For further information on Nature Portfolio policies, see our Editorial Policies and the Editorial Policy Checklist.

Statistics

For all statistical analyses, confirm that the following items are present in the figure legend, table legend, main text, or Methods section.

n/a	Confirmed	
☐	☒	The exact sample size (*n*) for each experimental group/condition, given as a discrete number and unit of measurement
☐	☒	A statement on whether measurements were taken from distinct samples or whether the same sample was measured repeatedly
☐	☒	The statistical test(s) used AND whether they are one- or two-sided<br>*Only common tests should be described solely by name; describe more complex techniques in the Methods section.*
☒	☐	A description of all covariates tested
☐	☒	A description of any assumptions or corrections, such as tests of normality and adjustment for multiple comparisons
☐	☒	A full description of the statistical parameters including central tendency (e.g. means) or other basic estimates (e.g. regression coefficient) AND variation (e.g. standard deviation) or associated estimates of uncertainty (e.g. confidence intervals)
☐	☒	For null hypothesis testing, the test statistic (e.g. *F*, *t*, *r*) with confidence intervals, effect sizes, degrees of freedom and *P* value noted<br>*Give P values as exact values whenever suitable.*
☒	☐	For Bayesian analysis, information on the choice of priors and Markov chain Monte Carlo settings
☒	☐	For hierarchical and complex designs, identification of the appropriate level for tests and full reporting of outcomes
☐	☒	Estimates of effect sizes (e.g. Cohen's *d*, Pearson's *r*), indicating how they were calculated

Our web collection on statistics for biologists contains articles on many of the points above.

Software and code

Policy information about availability of computer code

Data collection	Behavioral data collection used commercial software, including Noldus Ethovision XT, FLIR Integrated Imaging Solutions SpinView software, LabJack corporation LabJack U3-LV. Open source software including HandBrake for video conversion and Arduino for synchronization and optogenetic light delivery was also used. Calcium imaging acquisition used a combination of LabJack, Arduino, and the Inscopix Data Acquisition Software. Histological imaging was conducted using Olympus VS120 software as well as Olympus Fluoview for confocal image acquisition.
Data analysis	Calcium data processing was conducted in proprietary software from Inscopix (Mosaic). Open source constrained non-negative matrix factorization for endoscopic imaging (Zhou et al. 2017) was used for segmentation of calcium recordings in MATLAB (Mathworks) 2015b. Custom MATLAB scripts (MATLAB 2018-2020) were used for analysis of calcium events, fluorescence traces and event-related activity classification, and support vector machine decoding. This custom code has been uploaded to Zenodo (https://doi.org/10.5281/zenodo.10790736) and the source data uploaded as a Source Data file (see Data Availability and Code Availability sections). Analysis of behavioral data, as well as correlations, analyses of proportions were conducted using GraphPad Prism versions 7 to 9. Spectral clustering was conducted as described in Namboodiri et al. 2020 using Python 2.0 and the Scikit-learn package.

For manuscripts utilizing custom algorithms or software that are central to the research but not yet described in published literature, software must be made available to editors and reviewers. We strongly encourage code deposition in a community repository (e.g. GitHub). See the Nature Portfolio guidelines for submitting code & software for further information.

Data

Policy information about availability of data

All manuscripts must include a data availability statement. This statement should provide the following information, where applicable:

- Accession codes, unique identifiers, or web links for publicly available datasets
- A description of any restrictions on data availability
- For clinical datasets or third party data, please ensure that the statement adheres to our policy

The supporting data generated in this study are provided in the Source Data file.

Research involving human participants, their data, or biological material

Policy information about studies with human participants or human data. See also policy information about sex, gender (identity/presentation), and sexual orientation and race, ethnicity and racism.

Reporting on sex and gender	N/A
Reporting on race, ethnicity, or other socially relevant groupings	N/A
Population characteristics	N/A
Recruitment	N/A
Ethics oversight	N/A

Note that full information on the approval of the study protocol must also be provided in the manuscript.

Field-specific reporting

Please select the one below that is the best fit for your research. If you are not sure, read the appropriate sections before making your selection.

☒ Life sciences ☐ Behavioural & social sciences ☐ Ecological, evolutionary & environmental sciences

For a reference copy of the document with all sections, see nature.com/documents/nr-reporting-summary-flat.pdf

Life sciences study design

All studies must disclose on these points even when the disclosure is negative.

Sample size	No statistical methods were used to predetermine sample sizes. Our sample sizes for calcium imaging are similar to or exceed similar publications in the striatum (Parker et al. 2018, Markowitz et al. 2018) despite being from double-transgenic mice that are difficult to breed in large numbers. Similarly, sample size for indirect pathway optogenetic manipulations reflect what has been used in prior publications (Ottenheimer et al. 2020, Bolkan & Stone et al. 2022).
Data exclusions	Exclusion criteria were determined prior to experiments being conducted. For calcium imaging experiments, mice were excluded if upon histological assessment the GRIN lens was found to be outside of the central striatum. Similarly, for optogenetic experiments mice were excluded either if bilateral expression of ArchT or tdTomato was not present in the central striatum, or if the optic fibers were misplaced.
Replication	All experiments contained multiple biological replicates. Due to the difficulty of running large cohorts of in vivo freely moving calcium imaging, multiple cohorts were necessary to ensure our datasets were well powered. Thus, multiple cohorts of mice consistently reproduced our central observations.
Randomization	For calcium imaging experiments, all mice received identical surgical manipulations, thus no randomization was necessary. Both WT and KO mice were group housed except in the case of an aggressive cage-mate. For optogenetic experiments, a baseline lesion assessment was conducted and mice were assigned to either ArchT or eYFP in a balanced manner to ensure no baseline differences in grooming severity existed. For fluoxetine treatment experiments, all mice received fluoxetine in their drinking water thus no randomization was necessary.
Blinding	Due to manifestations of compulsive grooming being visually apparent during experiments, blinding of genotype was not always possible during behavioral experiments. Behavioral video (calcium imaging and optogenetics) was then assessed by blind observers. During calcium imaging analysis, the experimenter was not blind to genotype though all analyses were conducted uniformly across subjects and groups. For assessment of overlap of indirect pathway-projecting SPNs and expression of either Drd1a or Drd2, experimenters were blind to which pseudocolored image reflected which gene was being detected.

Reporting for specific materials, systems and methods

We require information from authors about some types of materials, experimental systems and methods used in many studies. Here, indicate whether each material, system or method listed is relevant to your study. If you are not sure if a list item applies to your research, read the appropriate section before selecting a response.

Materials & experimental systems

n/a	Involved in the study
☐	☒ Antibodies
☒	☐ Eukaryotic cell lines
☒	☐ Palaeontology and archaeology
☐	☒ Animals and other organisms
☒	☐ Clinical data
☒	☐ Dual use research of concern
☒	☐ Plants

Methods

n/a	Involved in the study
☒	☐ ChIP-seq
☒	☐ Flow cytometry
☒	☐ MRI-based neuroimaging

Antibodies

Antibodies used

Mouse α-Cre recombinase primary antibody (1:1500, MAB3120, Millipore).

Biotin-SP (long spacer) AffiniPure™ Goat Anti-Mouse IgG, $F(ab')_2$ fragment specific (1:250 Jackson ImmunoResearch - RRID: AB_2338565, Code: 115-065-072)

Cy™3 Streptavidin (1:250 Jackson ImmunoResearch - RRID: AB_2337244, Code: 016-160-084)

Validation

All antibodies used included validation conducted by the supplier as well as many peer-reviewed publications:

https://www.emdmillipore.com/US/en/product/Anti-Cre-Recombinase-Antibody-clone-2D8,MM_NF-MAB3120

https://www.jacksonimmuno.com/catalog/products/115-065-072

https://www.jacksonimmuno.com/catalog/products/016-160-084

Animals and other research organisms

Policy information about studies involving animals; ARRIVE guidelines recommended for reporting animal research, and Sex and Gender in Research

Laboratory animals

We used both male and female mice (4-8 months old at time of initial calcium or optogenetic surgery) for all studies. All mice were back-crossed to a full C57BL/6 background. Individual strains used include: Sapap3-WT and -KO mice (derived from a colony initially established by Dr. Guoping Feng's group at MIT). Drd1a-Cre (Tg(Drd1-cre)EY262Gsat/Mmucd; MMRRC: 017264), A2a-Cre (B6.FVB(Cg)-Tg(Adora2a-cre)KG139Gsat/Mmucd; MMRRC: 036158).
Double-transgenic (Drd1a-Cre+/- or A2a-Cre+/- and Sapap3+/+ or Sapap3-/-) were generated by breeding Sapap3 heterozygous (+/-) mutants with heterozygous Cre mice.

Wild animals

No wild animals were used for these experiments.

Reporting on sex

Male and female Sapap3-KO and wildtype littermates were used (see figure legends for numbers used in each experiment).

Figure 1) Sapap3-KO mice (n=11, 6 male / 5 female) - Sapap3-WT mice (n=8, 5 male / 3 female)
Figure 2) Sapap3-KO (n=6: 4 male / 2 female) - Sapap3-WT (n=8: 3 male / 5 female) mice
Figure 3) Sapap3-KO (n=8: 6 male/ 2 female) - Sapap3-WT (n=7: 5 male/ 2 female) mice
Figure 4) Sapap3-KOs expressing tdTomato control virus (n=7, 2 male / 5 female) - Sapap3-KO mice expressing ArchT (n=6, 2 male,4 female)
Figure 5) Sapap3-KOs (n=6: 6 female mice)

Field-collected samples

No field-collected samples were used for these experiments.

Ethics oversight

All procedures were carried out in accordance with the guidelines for the care and use of laboratory animals from the NIH and with approval from the University of Pittsburgh Institutional Animal Care and Use Committee (IACUC)

Note that full information on the approval of the study protocol must also be provided in the manuscript.

Plants

Seed stocks	N/A
Novel plant genotypes	N/A
Authentication	N/A
